# Dosimetric evaluation of skin collimation with tungsten rubber for electron radiotherapy: A Monte Carlo study

**DOI:** 10.1002/acm2.13210

**Published:** 2021-02-26

**Authors:** Kazuki Wakabayashi, Hajime Monzen, Mikoto Tamura, Kenji Matsumoto, Yoshiki Takei, Yasumasa Nishimura

**Affiliations:** ^1^ Department of Medical Physics Graduate School of Medical Sciences Kindai University Osaka Japan; ^2^ Department of Central Radiology Kindai University Hospital Osaka Japan; ^3^ Department of Radiation Oncology Faculty of Medicine Kindai University Osaka Japan

**Keywords:** electron radiotherapy, skin collimation, small‐field dosimetry, tungsten rubber

## Abstract

**Purpose:**

Skin collimation provides a sharp penumbra for electron beams, while the effect of bremsstrahlung from shielding materials is a concern. This phantom study was conducted to evaluate the safety and efficacy of a real‐time variable shape rubber containing‐tungsten (STR) that can be placed on a patient’s skin.

**Methods:**

Electron beam profiles were acquired with the STR placed on a water‐equivalent phantom and low melting‐point alloy (LMA) placed at the applicator according to commonly used procedures (field sizes: 20‐ and 40‐mm diameters). Depth and lateral dose profiles for 6‐ and 12‐MeV electron beams were obtained by Monte Carlo (MC) simulations and were benchmarked against film measurements. The width of the off‐axis distance between 80% and 20% doses (*P*_80‐20_) and the maximum dose were obtained from the lateral dose profiles. Bremsstrahlung emission was analyzed by MC simulations at the depth of maximum dose (*R*_100_).

**Results:**

The depth dose profiles calculated by the MC simulations were consistently within 2% of the measurements. The *P*_80‐20_ at *R*_100_ for 20‐ and 40‐mm diameters were 4.0 mm vs. 7.6 mm (STR vs. LMA) and 4.5 mm vs. 9.2 mm, respectively, for the 6‐MeV electron beam with 7.0‐mm‐thick STR, and 2.7 mm vs. 5.6 mm and 4.5 mm vs. 7.1 mm, respectively, for the 12‐MeV electron beam with 12.0‐mm‐thick STR. A hotspot was not observed on the lateral dose profiles obtained with the STR at *R*_100_. The bremsstrahlung emission under the region shielded by the STR was comparable to that obtained with the LMA, even though the STR was placed on the surface of the phantom.

**Conclusions:**

Skin collimator with STR provided superior dosimetric characteristics and comparable bremsstrahlung emission to LMA collimator at the applicator. STR could be a new tool for the safe and efficient delivery of electron radiotherapy.

## 1 INTRODUCTION

In electron radiotherapy, lead and low melting‐point alloy (LMA) are widely used to shape the irradiation field because of their excellent shielding ability against electron beams.^1^, ^2^, ^3^ However, the shortcoming of shielding materials made of lead or LMA equivalent includes toxicity to the human body.^4^, ^5^ In Japan, lead and LMA are commonly placed at the applicator to shape the irradiation field of the electron beam.^6^ However, an irradiation field shaped at the applicator has the disadvantage of a large penumbra compared with that shaped by a skin collimator.^7^

In a previous study, we reported that shielding materials containing tungsten, such as tungsten functional paper (TFP)^8^, ^9^, ^10^, ^11^, ^12^, ^13^ and tungsten‐containing rubber (TCR),^14^, ^15^, ^16^ can provide sufficient radiation shielding against therapeutic electron beams and diagnostic x‐rays and γ‐rays. In electron therapy, TCR offers the advantages of being nontoxic to the human body and being easy to shape the irradiation field. Recently, we developed real‐time variable shape rubber containing‐tungsten (STR) (Hayakawa Rubber Co., Ltd. Hiroshima, Japan) and demonstrated that the transmission rates reached plateau values at STR with thicknesses of ≥7.0 and ≥12.0 mm for 6‐ and 12‐MeV electron beams, respectively.^16^ A major advantage of the STR is that its shape can be changed by hand at a temperature of approximately 60°C, with it then maintaining this shape at room and body temperatures.^16^ A dynamic mechanical analysis showed that the ratio of viscosity to elasticity was 1.016 at the temperature of approximately 60°C, which is higher than the value of 0.6 required for shaping by hand.^16^ Therefore, the STR has the potential to facilitate flexible and immediate shaping of the irradiation field of electron beams.

Skin collimation is expected to produce a sharper penumbra^17^ and provide higher quality dose distribution in small‐field electron beam therapy.^18^, ^19^ On the other hand, the effect of bremsstrahlung from the shielding material is a concern by skin collimation.^11^ Kamomae et al. reported a slight increase in photon components at the exit plane of TFP as a chest wall protective disc in intraoperative electron radiotherapy for breast cancer treatment.^12^ However, it remains unclear whether the effect of bremsstrahlung from skin collimation is greater than that of shielding by conventional applicators in treatment for superficial lesions. Furthermore, for small‐field electron beams, the depth of the maximum dose and the sharpness of the dose fall‐off are different from those of a broad beam distribution because of the loss of lateral scatter equilibrium (LSE).^19^ Therefore, the dosimetric characteristics of small‐field electron beams for skin collimation with STR must be clarified.

The purpose of this study was to evaluate the occurrence of bremsstrahlung to demonstrate the safe delivery of electron radiotherapy using an STR skin collimator, making comparisons with a conventional LMA collimator placed at the applicator. Monte Carlo (MC) simulations were used for these evaluations. In addition, lateral dose profiles and percent depth doses (PDDs) were also examined to investigate the dose distribution of small‐field electron beams shaped by STR.

## 2 MATERIALS AND METHODS

### 2.1 Experimental setup

**Fig. 1:**
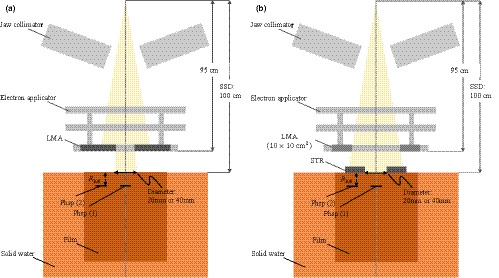
Schematic diagrams of the measurement geometry for (a) LMA and (b) STR. The thickness of the STR was 7.0‐ and 12.0‐mm for 6‐ and 12‐MeV electron beams. The same geometry was reproduced for the Monte Carlo (MC) simulation, and phase space files were used as the source. Details of the phase spaces used for the MC simulation of spectral distribution analysis are described in section *2.3*. LMA, Low melting‐point alloy; STR, Real‐time variable shape rubber containing‐tungsten, SSD, Source to surface distance.

Measurements of PDDs and lateral dose profiles in a water‐equivalent solid water phantom (GAMMEX, Wisconsin, USA) were performed with Gafchromic EBT3 films (ISP, Wayne, NJ, USA) for small‐field electron beams shaped with LMA or STR (Fig. 1). To shape the irradiation field, the LMA was placed at the applicator, whereas the STR was set on the surface of the solid water phantom. The density of the LMA was 9.8 g/cm^3^ and the element ratio (wt%) was In: 2.0%, Sn: 20.0%, Pb: 32.0%, and Bi: 46.0%. The density of the STR was 7.3 g/cm^3^ and the element ratio (wt%) was C: 5.5%, H: 0.9%, O: 1.4%, and W: 92.2%. The characteristics of the STR were described in detail in a previous report^16^. Circular irradiation fields of 20‐ and 40‐mm diameter were shaped using either LMA or STR at a source to surface distance (SSD) of 100 cm. The thickness of the LMA was 14.0 mm, and that of STR was 7.0‐ and 12.0‐mm for 6‐ and 12‐MeV electron beams, respectively. Electron beams with nominal energies of 6‐ and 12‐MeV were generated with a linear accelerator (Linac) (TrueBeam; Varian Medical Systems, Inc., Palo Alto, CA, USA). The size of the electron applicator was 10 cm × 10 cm, and 200 monitor units were set up for each film irradiated.

A dose calibration curve for the EBT3 film was prepared by irradiating the film with doses from 0.0 to 3.0 Gy, with an irradiation field size of 10 cm × 10 cm at the depth of the maximum dose in a solid water phantom. The exposed films were scanned using an ES‐G10000 (Epson Corp., Nagano, Japan) with a resolution of 72 dots‐per‐inch in 48‐bit RGB color images. All film measurements were analyzed from the red channel using ImageJ version 1.52a (National Institutes of Health, Bethesda, MD).

### 2.2 Measurement of the dose profiles

The film was set up parallel to the beam central axis (CAX) for PDDs and lateral dose profiles measurements. The lateral dose profiles at the surface were measured by arranging the film so that it was perpendicular to the beam axis at the phantom surface. The PDDs were normalized to the maximum dose. The depths of the maximum dose (*R*_100_), 90% dose (*R*_90_), 80% dose (*R*_80_), and photon contamination dose (*D_x_*; beyond the maximum range of electrons^19^) were obtained from the PDD curves. The lateral dose profiles at each depth were normalized by the dose at the CAX. The PDDs and lateral dose profiles of LMA and STR were compared for each field size and electron energy. The maximum doses within the irradiated field and the penumbra (as the width of the off‐axis distance from 80% to 20% dose levels, *P*_80‐20_) were evaluated at the surface, *R*_100_, *R*_90_, and *R*_80_.^16^

### 2.3 Monte Carlo simulations

The particle and heavy ion transport code system (PHITS) (version 2.74, Japan Atomic Energy Agency, Tokai, Japan) was used to simulate the behavior of the electron beam. PHITS can deal with the transport of nearly all particles (i.e., neutrons, protons, heavy ions, photons, and electrons) over wide energy ranges in three‐dimensional modeling systems.^20^ The Electron Gamma Shower version 5 (EGS5) algorithm was used for electron and photon transport in this study. The calculation parameters included a cut‐off for electron and positron kinetic energy of 0.1 MeV, photon cut‐off energy of 0.01 MeV, and 1.0 × 10^8^ source electrons. The number of particle histories was determined so that the statistical uncertainties evaluated as the standard error would be within ±1%, ±2%, and ±2% for each PDD, lateral dose profile, and spectral distribution, respectively.^9^ In this study, a simulation was performed using phase space files because the in‐head information of the TrueBeam linac is not disclosed. The phase space files for the TrueBeam linac were provided by the vendor in an International Atomic Energy Agency (IAEA) compatible format, and the details of these files are described by Rodrigues et al.^21^ The geometries in the MC simulations reproduced the experimental setups shown in Fig. 1. The dose grid sizes used for the calculation of the PDDs and lateral dose profiles were 5 mm × 5 mm × 1 mm and 2 mm × 5 mm × 2 mm, respectively. Spectral distributions were derived for (1) the CAX phase space and (2) the phase space under the shielded area which was defined as 25 mm outside of the irradiation field edge at *R*_100_, as shown in Fig. 1.

## 3 RESULTS

### 3.1 Measurement of the dose profile

**Fig. 2:**
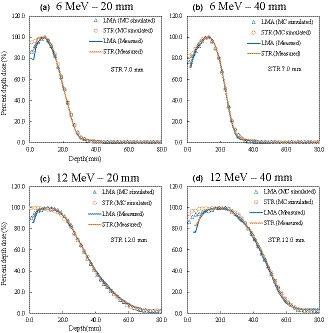
MC‐simulated (points) and measured (solid lines) PDDs for low melting‐point alloy (LMA) and 7.0‐ and 12.0‐mm‐thick real‐time variable shape rubber containing‐tungsten (STR) for 6‐ (a and b) and 12‐MeV (c and d) electron beams for the 20‐ and 40‐mm diameters. In each case, the profiles have been normalized to the maximum dose.

**Fig. 3:**
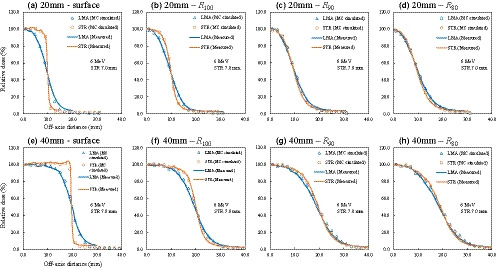
MC‐simulated (points) and measured (solid lines) lateral dose profiles for low melting‐point alloy (LMA) and 7.0‐mm‐thick real‐time variable shape rubber containing‐tungsten (STR) for 6‐MeV electron beams at the surface (a and e), *R*_100_ (b and f), *R*_90_ (c and g), and *R*_80_ (d and h) for the 20‐ and 40‐mm diameters. The lateral dose profiles at each depth were normalized by the dose at the central axis of the beam.

**Fig. 4:**
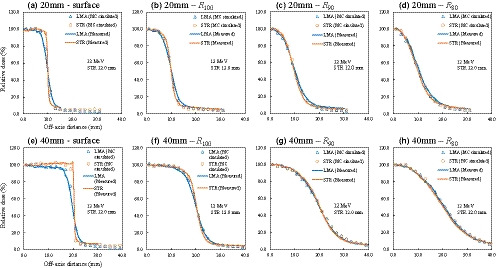
MC‐simulated (points) and measured (solid lines) lateral dose profiles for low melting‐point alloy (LMA) and 12.0‐mm‐thick real‐time variable shape rubber containing‐tungsten (STR) for 12 MeV electron beams at the surface (a and e), *R*_100_ (b and f), *R*_90_ (c and g), and *R*_80_ (d and h) for the 20‐ and 40‐mm diameters. The lateral dose profiles at each depth were normalized by the dose at the central axis of the beam.

The measured PDDs and lateral dose profiles are shown in Figs. 2, 3, 4. Table 1 shows the dosimetric characteristics obtained from the PDD curves (Fig. 2) for LMA and STR with each electron energy and field size. The PDDs for STR (7.0‐ and 12.0‐mm thicknesses with 6‐ and 12‐MeV electron beams) were almost equal to those for the LMA, with dose differences within 2% at *R*_100_, *R*_90_, and *R*_80_, and within 0.2% at *D_x_*. Figs. 3 and 4 show the lateral dose profiles of LMA and 7.0‐ and 12.0‐mm‐thick STR for the 20‐ and 40‐mm diameters with 6‐ and 12‐MeV electron beams. Table 2 shows the dosimetric characteristics of the penumbras from the lateral dose profiles with LMA and STR. The *P*_80‐20_ values at *R*_100_ for a 6‐MeV electron beam were 7.6 mm with LMA and 4.0 mm with 7.0‐mm‐thick STR for the 20‐mm‐diameter, whereas for the 40‐mm‐diameter, they were 9.2 mm and 4.5 mm, respectively. With a 12‐MeV electron beam, the corresponding values for the 20‐mm‐diameter were 5.6 mm with LMA and 2.7 mm with 12.0‐mm‐thick STR, and 7.1 mm and 4.5 mm for the 40‐mm‐diameter. The *P*_80‐20_ values of the STR were smaller than those of the LMA. The lateral dose profile with STR at the surface showed a high dose at the edge of the irradiation field with both energies and the 40‐mm‐diameter (Figs. 3e and 4e). The maximum doses at the surface for the 20‐ and 40‐mm diameters were 101.9% and 104.5% for the STR with the thickness of 7.0 mm in 6‐MeV electron beam (Fig. 3a and 3e), respectively, and 101.0% and 105.4% for the STR with the thickness of 12.0 mm in 12‐MeV electron beam (Fig. 4a and 4e). However, no high dose region was observed in the STR lateral dose profile at *R*_100_ or more. For the STR with the thickness of 7.0 mm in 6‐MeV electron beam, the maximum doses at *R*_100_ (Fig. 3b and 3f) for 20‐ and 40‐mm diameters were 100.4% and 100.5%, respectively, whereas for the STR with the thickness of 12.0 mm in 12 MeV electron beam, the corresponding values were 100.1% and 100.2%, respectively (Fig. 4b and 4f).

**Table 1:** Dosimetric characteristics of PDDs for 20‐ and 40‐mm diameters with LMA and 7.0‐ and 12.0‐mm‐thick STRs for 6‐ and 12‐MeV electron beams.

Energy (MeV)	Shielding material	20 mm diameter				40 mm diameter			
		*R*_100_ (mm)	*R*_90_ (mm)	*R*_80_ (mm)	*D*_x_ (%)	*R*_100_ (mm)	*R*_90_ (mm)	*R*_80_ (mm)	*D*_x_ (%)
6	LMA	8.7	12.9	15.3	2.4	12.6	17.3	19.0	2.3
	STR	8.5	13.4	15.4	2.3	12.5	17.1	19.2	2.5
12	LMA	9.3	22.1	25.5	3.9	17.8	32.1	36.8	3.9
	STR	8.4	21.2	25.2	3.9	17.7	32.2	37.5	3.9

Abbreviations: LMA, Low melting‐point alloy; STR, Real‐time variable shape rubber containing‐tungsten; PDD, percent depth doses.

**Table 2:** Penumbras (mm) at the surface, *R*_100_, *R*_90_, and *R*_80_ in lateral dose profiles for 20‐ and 40‐mm diameters with LMA and 7.0‐ and 12.0‐mm‐thick STRs for 6‐ and 12‐MeV electron beams.

Energy (MeV)	Shielding material	20 mm diameter				40 mm diameter			
		Surface	*R*_100_	*R*_90_	*R*_80_	Surface	*R*_100_	*R*_90_	*R*_80_
6	LMA	6.1	7.6	9.5	10.3	7.5	9.2	11.7	13.0
	STR	0.3	4.0	6.1	7.1	0.3	4.5	8.1	10.2
12	LMA	3.4	5.6	8.2	9.2	4.1	7.1	12.8	15.5
	STR	0.4	2.7	6.1	8.7	0.5	4.5	10.8	15.0

Abbreviations: LMA, Low melting‐point alloy; STR, Real‐time variable shape rubber containing‐tungsten.

### 3.2 Monte Carlo simulation modeling

The PDDs (Fig. 2) and lateral dose profiles with MC simulation (Figs. 3 and 4) were in close agreement with the measurements. The differences of *R*_100_, *R*_90_, and *R*_80_ between the MC simulation and measurement of the PDD were within 2%, with the exception of data from shallow depths. For the lateral dose profiles, the distance to agreement (DTA) between the MC simulation and measurement in the penumbra region was less than 2 mm.

### 3.3 Spectral distributions with Monte Carlo simulations

**Fig. 5:**
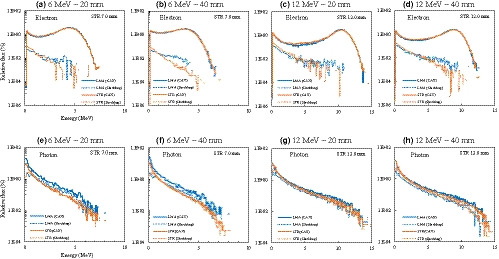
Spectral distributions of the electron and photon components at the central axis of the beam (CAX) and the under the shielded area (Shielding), for low melting‐point alloy (LMA) and 7.0‐ and 12.0‐mm‐thick real‐time variable shape rubber containing‐tungsten (STR) with 6‐ and 12‐MeV electron beams at *R*_100_ for the 20‐ and 40‐mm diameters. The electron and photon components were normalized by the total number of each particle in the CAX. The first and second rows show the electron (a‐d) and photon (e‐h) components, respectively.

Figure 5 shows the results of the spectral distributions at *R*_100_. The electron and photon components of LMA and STR were normalized by the total number of each particle in the CAX. For 6‐MeV electron beams, the peak electron energies of both LMA and STR at the CAX were 4.25 MeV and 3.75 MeV for the 20‐ and 40‐mm diameters, respectively, whereas the corresponding values for 12‐MeV electron beams were 10.40 MeV and 8.75 MeV, respectively. The LMA and STR electron distributions at the CAX (Fig. 5a–5d) were correlated for each energy and field size; however, the electron distributions under the shielded area with STR (Fig. 5a–5d) were comparable or lower than those with LMA. The photon components at the CAX and under the shielded area (Fig. 5e–5h) with STR were comparable to or lower than those with LMA, except for the low energy region (<3 MeV) for the 12‐MeV electron beam under the shielded area. In this region, the difference in photon components between STR and LMA was at most 2%.

## 4 DISCUSSION

In this study, we evaluated the dosimetric characteristics of small‐field electron beams for skin collimation with STR, and the electron radiotherapy with the STR placed on the surface of a patient could be performed safely and effectively.

The PDDs (Fig. 2 and Table 1) were almost equal between LMA and STR, which indicates that the dosimetric characteristics of the depth dose profile were not affected by the shielding material and position. This result was in agreement with our previous report^16^ demonstrating that the method was effective even for a small‐field. The STR also provided a sharp penumbra by shielding close to the patient, which has advantages when protecting adjacent critical structures from small‐field electron therapy. The maximum doses around the edge of the irradiation field with STR at the phantom surface were higher than those with LMA (Figs. 3a, 3e, 4a, and 4e) because of the effects of electron scattering from the edge of the STR.^17^ However, for a 40‐mm diameter, the relative magnitudes of those high doses were approximately 80% and 90% of the prescribed doses with 6‐ and 12‐MeV electron beams, because the PDD values at the surface were approximately 75% and 85%, as shown in Fig. 2.^16^ In addition, even though thick STR is needed (e.g., 12 mm), it is easy to cut pieces out of the material by hand, and it provides a sharp dose falloff at the edge of the irradiation field. Although a variable thickness, especially at the edges, may cause a high dose due to penetration and scatter,^12^, ^17^ in this case, the high dose was at most 90% of the prescribed dose. Therefore, we conclude that manual shaping of STR was not an issue in relation to beam divergence. The high dose region decreased as the depth increased because the scattered electrons were low energy.^17^

The spectral distribution analysis showed that the generation of bremsstrahlung from the shielding material^12^ was not a clinical concern. The bremsstrahlung from the STR was lower or comparable to that from the LMA, except for the low energy region (<3 MeV) for the 12‐MeV electron beam under the shielded area. However, with the STR, the increase in photon components within this energy region was at most 2% (Fig. 5e–5h), while the standard error of the MC simulation was ±2%, indicating the difference was not significant. Furthermore, photon contamination was comparable between STR and LMA, as shown in Table 1. On the other hand, the electron distribution of STR correlated with LMA in the CAX, and was comparable to or less than that of LMA under the shielded area; hence, STR could provide the safe delivery in clinical practice.

The results of our experiments were consistent with those of Perez et al.^22^ who reported results for lead‐based shielding. As the placement of the shielding material directly on the skin reduces the spread of the penumbra, we expect that STR will reduce the additional margin and exposure to normal tissues in comparison with previous methods involving the placement of LMA at the applicator. The STR also has the advantage that it can be shaped in real‐time, thereby facilitating quick and individualized electron radiotherapy for each patient, such as for the treatment of keloid scars, where it is important to start the treatment within 7 h after excision to decrease recurrence rates.^23^ A real‐time shaping procedure could proceed as follows: (a) the STR wrapped in clingfilm is heated in a 600 W microwave oven for 1 min, (b) it is rolled out to a sheet of uniform thickness, (c) the irradiation field is cut out of the STR using a shaped cutter or scissors. In addition, STR can adapt to the patient’s respiratory motion and the rounded contours of the patient’s body; it can be placed directly on the body surface because it is nontoxic.^16^ STR could be useful for lesions of the ear, nose, breast, belly, and those around the eye, and it could also be applied in intraoperative electron radiotherapy,^12^ in electron grid therapy,^9^ and as an eye shield^24^ instead of conventional devices.

## 5 CONCLUSION

The STR skin collimator provided superior dosimetric characteristics and comparable the occurrence of bremsstrahlung to the conventional LMA collimator at the applicators. STR, which can be placed on the surface of the patient, is a promising new tool to aid the safe and effective delivery of small‐field electron radiotherapy.

## Conflict of Interest

Hajime Monzen received a research donation from Hayakawa Rubber Co., Ltd.

## Authors’ contributions

Conceptualization: K Wakabayashi, H Monzen, M Tamura. Funding acquisition: H Monzen. Methodology: K Wakabayashi, M Tamura. Investigation: K Wakabayashi, M Tamura, K Matsumoto. Data curation: K Wakabayashi, M Tamura, Y Takei. Writing – original draft: K Wakabayashi, H Monzen, M Tamura. Writing – review & editing: H Monzen, M Tamura, K Matsumoto, Y Takei, Y Nishimura.
